# Herpesviruses, polyomaviruses, parvoviruses, papillomaviruses, and anelloviruses in vestibular schwannoma

**DOI:** 10.1007/s13365-023-01112-8

**Published:** 2023-03-01

**Authors:** Maria K. Jauhiainen, Ushanandini Mohanraj, Martin Lehecka, Mika Niemelä, Timo P. Hirvonen, Diogo Pratas, Maria F. Perdomo, Maria Söderlund-Venermo, Antti A. Mäkitie, Saku T. Sinkkonen

**Affiliations:** 1 Department of Otorhinolaryngology - Head and Neck Surgery, Helsinki University Hospital and University of Helsinki, POB 263, 00029 Helsinki, Finland; 2 Department of Virology, University of Helsinki, Helsinki, Finland; 3 Faculty of Medicine, Research Program in Systems Oncology, University of Helsinki, Helsinki, Finland; 4 Department of Neurosurgery, Helsinki University Hospital and University of Helsinki, Helsinki, Finland; 5 Faculty of Medicine, The Doctoral Programme in Clinical Research, University of Helsinki, Helsinki, Finland; 6 Department of Electronics, Telecommunications and Informatics and Institute of Electronics and Informatics Engineering of Aveiro, University of Aveiro, Aveiro, Portugal

**Keywords:** Vestibular schwannoma, Viral etiology, Herpesvirus, Polyomavirus, Parvovirus, Anellovirus

## Abstract

Etiology of vestibular schwannoma (VS) is unknown. Viruses can infect and reside in neural tissues for decades, and new viruses with unknown tumorigenic potential have been discovered. The presence of herpesvirus, polyomavirus, parvovirus, and anellovirus DNA was analyzed by quantitative PCR in 46 formalin-fixed paraffin-embedded VS samples. Five samples were analyzed by targeted next-generation sequencing. Viral DNA was detected altogether in 24/46 (52%) tumor samples, mostly representing anelloviruses (46%). Our findings show frequent persistence of anelloviruses, considered normal virome, in VS. None of the other viruses showed an extensive presence, thereby suggesting insignificant role in VS.

**Supplementary Information:**

The online version contains supplementary material available at 10.1007/s13365-023-01112-8.

## Introduction

Vestibular schwannoma (VS) arises from the eight cranial nerve (CNVIII) and can occur either sporadically or as a result of neurofibromatosis 2 (NF2) syndrome. The etiology of sporadic VS is unknown. Typical risk factors for malignancies, such as tobacco smoking and alcohol consumption, do not seem to play a significant role in this entity. The only identified risk factors are medical irradiation during early life and exposure to radiation hazards (Preston et al. 2002; Ron et al. 1988; Schneider et al. 2008). Studies have shown possible links of sporadic VS to certain genes (De Vries et al. 2015); however, the trigger for a tumor-forming cascade is unknown. A possible microbial etiology has been studied less.

Viruses and other microbes are estimated to cause 15% of all cancers worldwide (Plummer et al. 2016). This subject is even more topical than ever before as the rapid evolution in virus research has brought attention to newly discovered viruses. The role of viral infections as an etiological factor in intracranial tumors has previously been proposed. The prevalence of cytomegalovirus (CMV) in glioblastoma was as much as 84% among 1653 tumors (Rahman et al. 2018). Merkel cell polyomavirus (MCPyV) has been found in several head and neck tumors, including neurofibromas (1/3, 33%) and schwannomas (2/12, 17%) (Tanio et al. 2015).

Given this background, and the potential exposure of the CNVIII to middle ear viruses and other microbes, it is intriguing to speculate on a potential viral etiology of VS. A few studies have been published regarding this topic. Bhimrao et al. (2015) investigated the prevalence of certain herpesviruses in tissue microarray samples of sporadic VS and neurofibromatosis-related schwannomas, but none were detected. Håvik et al. (2018) applied ViroChip microarray (*n* = 15 patients), RT-PCR for human endogenous retrovirus K (HERV-K) and herpes simplex virus-1 and -2 (HSV-1, -2) RNA (*n* = 46), and whole genome sequencing (*n* = 2). HERV-K was detected, but other findings remained modest. The ViroChip used in the study was published 20 years ago.

The DNA-virus families in focus in the present study (*Herpesviridae*, *Parvoviridae*, *Polyomaviridae*, *Papillomaviridae*, and *Anelloviridae*) comprehend oncogenic and cancer-associated viruses, as well as viruses with oncolytic abilities (Beral et al. 1990; Chang et al. 1994; Feng et al. 2008; Gissmann et al. 1984; Kreuter et al. 2018; Nüesch et al. 2012; Syrjänen et al. 1983; Väisänen et al. 2019). Within the total of nine viruses of the human herpesvirus family, human herpesvirus 8 (HHV-8) causes Kaposi ‘s sarcoma (Beral et al. 1990; Chang et al. 1994), and Epstein-Barr virus (EBV) is strongly associated with, e.g., Burkitt’s lymphoma and nasopharyngeal carcinoma (Henle et al. 1970). HSV-1 and -2 and varicella zoster virus (VZV), on the other hand, are neurotropic and widespread, but without clear association with malignancies.

Human polyomaviruses comprise a group of 15 viruses, the majority discovered during the last decade, and most with a high seroprevalence among humans. MCPyV causes Merkel cell carcinoma in humans; however, the association of polyomaviruses to other tumors is still unknown (Csoboz et al. 2020; De-Thé et al. 1978; Feng et al. 2008). Among parvoviruses, many viruses have been discovered in the past two decades: human bocaviruses 1–4 (HBoV 1–4), parvovirus 4 (Parv4), and the protoparvoviruses bufaviruses (BuV), cutaviruses (CuV), and tusaviruses (TuV; (Söderlund-Venermo 2019). HBoV1 causes pediatric respiratory tract infections, whereas CuV has been associated with cutaneous T cell lymphoma (Kreuter et al. 2018; Phan et al. 2016; Qiu et al. 2017; Väisänen et al. 2019). However, the clinical roles of the other recently discovered viruses need further investigation.

Anelloviruses encompass a broad scale of viruses, including the ubiquitous torque teno viruses (TTV). No causal association to any disease has been made; thereby, they are considered a part of the normal human virome.

The unknown etiology of VS, with abundant virus load in the anatomical proximity constituting a potential trigger, formed the motivation for the present study. Quantitative PCR (qPCR) is the gold standard for virus detection due to its high sensitivity and specificity, whereas next-generation sequencing (NGS) enables wider multiplex-screening and analysis of the virus sequence. We performed a retrospective cross-sectional study to evaluate the prevalence of viral DNAs in VS by qPCRs and NGS, targeting many recently discovered virus entities not studied before.

## Materials and methods

### Ethics

A research ethics committee approval (HUS/31/07.03.2019) and an institutional research permission (HUS/332/2019) were obtained at the Helsinki University Hospital.

### Patient characteristics and clinical specimens

The patients were treated at the Departments of Neurosurgery and Otorhinolaryngology – Head and Neck Surgery, Helsinki University Hospital, Helsinki, Finland, between 2008 and 2018. Altogether 46 FFPE tumor samples from 46 patients were collected from the Helsinki Biobank (permission no. §73/15.05.2019, HUS/118/2019). The clinical information was recorded from hospital charts.

There were 30 females (65%) and 16 males (35%). The mean age at operation was 52.8 years (median 53.1, range 32.3–80.9). Forty-four patients had sporadic tumors. One patient had bilateral VS suggestive of neurofibromatosis 2, and one patient had multiple meningiomas, although the genetic testing for neurofibromatosis was negative. Only six (13%) patients were known smokers, while the smoking status was unknown for 26 (57%) patients. The alcohol use was not recorded for 85% of the patients. Three (7%) patients used immunomodulatory drugs, and two (4%) patients had diabetes. VS had an extracanalicular extension in 44 (96%) cases. Radiation therapy (RT) was given altogether to six (13%) patients, but preoperatively only to one (2%) patient.

The samples were collected from FFPE tissue blocks as 2-mm punch biopsies in a PCR-sterile manner from the site of the tumor tissue — one sample for the qPCR and another from a nearby location for NGS. All samples were collected in 1.5 ml microcentrifuge tubes and stored until DNA extraction.

### DNA extraction

DNA was extracted using QIAamp DNA mini kit (Qiagen, Heiden, Germany) according to the manufacturer’s protocol, with slight modifications: paraffin removal was done twice and 40 µl of proteinase K was used. The DNA preps were eluted in 100 µl AE buffer and stored at − 20 °C.

Evaluation of DNA quality and human cell quantity was done by subjecting the samples to the reference gene *RNase*-P qPCR, as described earlier (Toppinen et al. 2015).

### Virus DNA detection by qPCR

The viruses analyzed with qPCR are listed in Supplemental Table 1. For the nine human viruses of *Herpesviridae*, a three-tube multiplex qPCR assay (HSV-1, HSV-2, and VZV; HHV-6A, HHV-6B, and HHV-7; EBV, cytomegalovirus (CMV), and HHV-8) was applied to target each virus (Pyöriä et al. 2020). For three virus members of *Polyomaviridae,* BK polyomavirus (BKPyV), JCPyV, and MCPyV, the LT genes were detected by singleplex qPCR assays (Dumoulin and Hirsch 2011; Goh et al. 2009). As for the members of *Parvoviridae,* parvovirus B19 (B19V) was detected and quantified with a pan-B19 qPCR, targeting the *NS1* genes of all three genotypes (Toppinen et al. 2015), whereas HBoV1-4 were detected by a multiplex qPCR, targeting the NS1 regions of all four bocaviruses (Kantola et al. 2010). CuV, BuV, and TuV were detected and quantified by a multiplex qPCR (Väisänen et al. 2019) targeting the NS1 region of BuV and VP2 regions of TuV and CuV. For TTV detection, the conserved untranslated region (UTR) was targeted by a multiplex qPCR, which detects most TTV types (Toppinen et al. 2020; Väisänen et al. 2022). All real-time qPCR assays were performed with AriaMx Realtime PCR System (Agilent Technologies, Santa Clara, CA).

**Table 1:** Different virus types detected with qPCR in 46 vestibular schwannoma samples

		**Viral DNA findings**	**Virus load (virus DNA/1000 000 cells)**
***Herpesviridae***	Human herpesvirus 7	1	2.54 × 10^1^
	Epstein –Barr Virus	1	8.75 × 10^0^
***Polyomaviridae***	Merkel cell polyomavirus	3	Mean 6.87 × 10^1^
***Parvoviridae***	Parvovirus B19	1	2.86 × 10^1^
***Anelloviridae***	Torque teno virus	21	1.08 × 10^4^

Molecular biology grade water was included in all PCR reactions as non-template control. Ten-fold diluted plasmids (10^1^–10^6^), containing each viral target amplicon, served as PCR standards and as positive controls. To prevent contamination, the master mix components, tumor samples, and plasmids were each handled in separate rooms. Laminar hoods were used when preparing and handling the samples and plasmids.

### Virus DNA detection by NGS

The viruses analyzed with targeted NGS are listed in Supplemental Table 1. The DNA was mechanically fragmented with a Covaris E220 with a target length of 200 nt. Subsequently, the libraries were prepared with the KAPA HyperPlus kit (Roche) using unique Dual Index Adapters (Roche). Targeted enrichment of the viral DNAs was performed using a custom panel of biotinylated RNA-probes covering the full length of the viral genomes (Arbor Biosciences) as described (Toppinen et al. 2020). Each sample was individually enriched via two rounds of hybridization, following the manufacturer recommendations for low input DNA (MyBaits v5 kit; Arbor Biosciences). The probes were 100 bp in length and designed with 2 × tiling. Kapa Universal Blockers (Roche) were used to block unspecific binding to the adapters during hybridization.

During library preparation and viral enrichment, the libraries were amplified 3 × 13–25 cycles. The clean-up steps were performed with 0.8 × KAPA HyperPure Beads (Roche). The enriched libraries were quantified with the KAPA Library Quantification Kit (Roche) using Stratagene 3005P qPCR System (Agilent) and pooled for sequencing on NovaSeq 6000 (S1, PE151 kit; Illumina).

### NGS analysis

The data analysis was done with TRACESPipeLite, a streamlined version of TRACESPipe (Pratas et al. 2020). TRACESPipeLite is fully automatic and provides for each reconstructed viral genome, the consensus sequences, breadth and depth coverage, and the associated profiles, among other information and quality controls. The paired-end reads were trimmed and collapsed with AdapterRemoval, cutting ambiguous bases at the 5′/3′ termini with quality scores below or equal to two. Reads shorter than 20 bases were discarded. FALCON-meta (Pratas et al. 2018) was used to find the highest similar reference from the NCBI viral database. The reads were aligned with BWA (Li and Durbin 2010) using a seed length of 1000 and a maximum diff of 0.01. Read duplicates were removed with SAMtools (Li et al. 2009) and the consensus sequences reconstructed with BCFtools (Li 2011). The coverage profiles were created with BEDtools (Quinlan 2014). When in low breadth coverage (< 15%), the individual reads were manually inspected and confirmed by BLAST. The pipeline is freely available, under MIT license, at https://github.com/viromelab/TRACESPipeLite, along with the code (included in the TRACESPipeLite repository).

## Results

### Presence of virus DNA by qPCR

The viruses analyzed with qPCR are listed in Supplemental Table 1. Altogether, 26 viral DNA findings were made by qPCR in 24/46 (52%) tumors (Table 1). One tumor had three viral DNA findings, and two tumors had two viral findings each. The viral loads ranged between 9 and 121,000 copies per 1 million cells (cpm). TTV DNA was detected in altogether 21/46 (46%) tumors, with viral loads of 1.38–8.91 × 10^4^, mean 1.08 × 10^4^ cpm. In 15/46 (33%) tumors, TTV PCR was positive in duplicate analysis. MCPyV was detected in 3/46 (7%) tumors. One tumor was positive for EBV, one tumor for B19V, and one tumor for HHV-7 DNA. Duplicate analysis performed for all these six tumor samples tested negative, probably due to low virus loads (mean 44.8, median 39.3, range 8.75–89.3 cpm). Sixteen out of 21 viruses from our screen were not detected in any sample. The two patients with probable NF2 tested both positive for TTV but negative for all other viruses. The human *RNaseP* served as evaluation of DNA quality and human cell quantity and its qPCR results varied between 10^2^ and 10^5^ copies/μl among all samples with a mean of 4.4 × 10^3^.

### Presence of virus DNA in NGS

The viruses analyzed with targeted NGS are listed in Supplemental Table 1. Tumor samples from five patients, previously analyzed with qPCR, were chosen for targeted enrichment and sequencing, four samples because of their viral DNA positivity (MCPyV, EBV, HHV-7) and one sample without any viral DNA by qPCR as a negative control. Also, one peripheral neural tissue sample was used as a control. Human endogenous retrovirus (HERV) served as an internal control, and all the six samples were positive for it. One tumor gave a single read for B19V DNA (one manually confirmed read). Apart from HERV and B19V, all the samples were DNA negative for all the other 40 tested viruses.

## Discussion

This study is an up-to-date qPCR and NGS screening to detect both well-known and recently discovered viruses (herpesviruses, polyomaviruses, parvoviruses, and papillomaviruses) in 46 VS. QPCR for 20 viruses of three virus families (herpesviruses, polyomaviruses, and parvoviruses; Supplemental Table 1) resulted in six positive virus DNA findings (MCPyV, EBV, B19V, HHV-7). MCPyV was detected in three (3/46, 6.5%) tumors, with approximately similar results of two in 12 schwannomas (17%) by Tanio et al (2015). A possible “hit-and-run” mechanism has been proposed for MCPyV (Prezioso et al. 2021), but in our study, we were unable to investigate such potential because of the lack of parallel lymph node samples. EBV, B19V, and HHV-7 were each detected once.

QPCR was supplemented with targeted NGS to both verify the findings, and to broaden the spectrum of screened viruses. Forty-one viruses of seven virus families (Supplemental Table 1) in five tumors, previously analyzed by qPCR, were investigated with targeted enrichment and sequencing. Only B19V DNA was detected in one tumor sample.

In addition, wide-spread anelloviruses, namely, TTVs, were screened by qPCR. Their DNA was found in 21/46 (46%) tumors. This was expected, since TTVs are considered normal flora (Arze et al. 2021). Given that TTV is actively replicating in healthy individuals and that its clinical significance has not yet been determined, the presence in VS is insufficient proof of association, not to mention causality.

QPCR offers a solid, sensitive base for virus screening. NGS with targeted enrichment is also well justified in virus research. We used a panel that includes 90% of the DNA viral families and majority of viruses with a clinical significance in humans (Supplemental Table 1). Whole genome sequencing has undeniably an even broader potential in the extent of screening, but its sensitivity for virus detection is low, and therefore, the approach with targeted viruses was preferred in this study. NGS overcomes the limitations of FFPE samples (low amount of DNA, DNA fragmentation, DNA deamination) that can potentially affect the performance of PCR.

QPCR and NGS results were largely consistent with each other, in that only limited amounts of virus DNAs were found. This is in accordance with previous studies (Bhimrao et al. 2015; Havik et al. 2018). The more recently identified viruses searched for in our study (bocaviruses, bufavirus, cutavirus, tusavirus, polyomaviruses) were not studied in the previous reports. HERV was detected in all our sequenced samples and serves as an internal control rather than a potential etiological factor.

In this study, we were unable to repeat our original qPCR results with duplicate qPCR analysis or detect the same viruses with NGS. This might be due to low virus load in combination with a second freeze–thaw round needed for NGS with possible degraded DNA. In addition, although FFPE samples have previously been shown to be a useful source for virus screening (Jauhiainen et al. 2021), it is possible that with this tumor type, larger samples and fresh tumor material would be beneficial as they may increase the amount of intact DNA and improve its quality (Mielonen et al. 2022).

When searching for possible viral etiology for VS, one has to bear in mind that the mere presence of virus DNA in a tissue sample does not indicate a causative role in disease development, and neither does its absence. A possible “hit-and-run” phenomenon may be involved as the trigger (Ferreira et al. 2021), but this would warrant more advanced techniques, not yet available, to be investigated.

## Conclusion

Based on the current and previous studies, it seems unlikely that herpesviruses, polyomaviruses, parvoviruses, and papillomaviruses play a significant etiological role in VS. Thus, the etiology of VS remains to be studied further.

## Supplementary Information

Below is the link to the electronic supplementary material.

Supplementary file1 (DOCX 135 KB)

## Data Availability

For possible data requests, the first Author should be contacted.

## References

[CR1] Arze CA, Springer S, Dudas G, Patel S, Bhattacharyya A, Swaminathan H, Brugnara C, Delagrave S, Ong T, Kahvejian A, Echelard Y, Weinstein EG, Hajjar RJ, Andersen KG, Yozwiak NL (2021). Global genome analysis reveals a vast and dynamic anellovirus landscape within the human virome. Cell Host Microbe.

[CR2] Beral V, Peterman TA, Berkelman RL, Jaffe HW (1990). Kaposi’s sarcoma among persons with AIDS: a sexually transmitted infection?. Lancet (london, England).

[CR3] Bhimrao SK, Maguire J, Garnis C, Tang P, Lea J, Akagami R, Westerberg BD (2015). Lack of association between human herpesvirus and vestibular schwannoma: analysis of 121 cases. Otolaryngol-Head Neck Surg : Official J Am Acad Otolaryngol-Head Neck Surg.

[CR4] Chang Y, Cesarman E, Pessin MS, Lee F, Culpepper J, Knowles DM, Moore PS (1994). Identification of herpesvirus-like DNA sequences in AIDS-associated Kaposi's sarcoma. Science (new York, NY).

[CR5] Csoboz B, Rasheed K, Sveinbjørnsson B, Moens U (2020) Merkel cell polyomavirus and non-Merkel cell carcinomas: guilty or circumstantial evidence? APMIS Journal of Pathology, Microbiology and Immunology 10.1111/apm.13019 31990105

[CR6] De Vries M, Van der Mey AGL, Hogendoorn PCW (2015) Tumor biology of vestibular Schwannoma: a review of experimental data on the determinants of tumor genesis and growth characteristics 10.1097/MAO.0000000000000788 26049313

[CR7] De-Thé G, Geser A, Day NE, Tukei PM, Williams EH, Beri DP, Smith PG, Dean AG, Bronkamm GW, Feorino P, Henle W (1978). Epidemiological evidence for causal relationship between Epstein-Barr virus and Burkitt’s lymphoma from Ugandan prospective study. Nature 274:756-61 10.1038/274756a0 210392

[CR8] Dumoulin A, Hirsch HH (2011) Reevaluating and optimizing polyomavirus BK and JC real-time PCR assays to detect rare sequence polymorphisms. J Clin Microbiol 49(4):1382–1388 10.1128/JCM.02008-10 PMC3122792 21325560

[CR9] Feng H, Shuda M, Chang Y, Moore PS (2008). Clonal integration of a polyomavirus in human Merkel cell carcinoma. Science (new York, NY).

[CR10] Ferreira DA, Tayyar Y, Idris A, McMillan NAJ (2021) A “hit-and-run” affair — a possible link for cancer progression in virally driven cancers. Biochim Biophys Acta Rev Cancer 18975(1) 10.1016/j.bbcan.2020.188476 33186643

[CR11] Gissmann L, Boshart M, Dürst M, Ikenberg H, Wagner D, Hausen HZ (1984) Presence of human papillomavirus in genital tumors. Invest Dermatol 83:S26–S28 10.1111/1523-1747.ep12281143 6330218

[CR12] Goh S, Lindau C, Tiveljung-Lindell A, Allander T (2009) Merkel cell polyomavirus in respiratory tract secretions. Emerg Infect Dis 15(3):489–491 10.3201/eid1503.081206 PMC2681127 19239773

[CR13] Havik AL, Bruland O, Aarhus M, Kalland KH, Stokowy T, Lund-Johansen M, Knappskog PM (2018). Screening for viral nucleic acids in vestibular schwannoma. J Neurovirol.

[CR14] Henle W, Henle G, Ho H, Burtin P, Cachin Y, Clifford P, De Schryver A, De-Thé G, Diehl V, Klein G (1970) Antibodies to Epstein-Barr virus in nasopharyngeal carcinoma, other head and neck neoplasms, and control groups. J Natl Cancer Inst 44(1):225–231 11515035

[CR15] Jauhiainen MK, Xu M, Pyoria L, Atula T, Aro K, Markkanen A, Haglund C, Hagstrom J, Makitie AA, Soderlund-Venermo M, Sinkkonen ST (2021). The presence of herpesviruses in malignant but not in benign or recurrent pleomorphic adenomas. Tumour Biol.

[CR16] Kantola K, Sadeghi M, Antikainen J, Kirveskari J, Delwart E, Hedman K, Söderlund-Venermo M (2010). Real-time quantitative PCR detection of four human bocaviruses. J Clin Microbiol.

[CR17] Kreuter A, Nasserani N, Tigges C, Oellig F, Silling S, Akgül B, Wieland U (2018). Cutavirus Infection in Primary Cutaneous B- and T-Cell Lymphoma. JAMA Dermatol.

[CR18] Li H (2011) A statistical framework for SNP calling, mutation discovery, association mapping and population genetical parameter estimation from sequencing data. Bioinformatics 27(21) 10.1093/bioinformatics/btr509 PMC3198575 21903627

[CR19] Li H, Durbin R (2010) Fast and accurate long-read alignment with Burrows-Wheeler transform 10.1093/bioinformatics/btp698 PMC2828108 20080505

[CR20] Li H, Handsaker B, Wysoker A, Fennell T, Ruan J, Homer N, Marth G, Abecasis G, Durbin R (2009) The sequence alignment/map format and SAMtools. Bioinformatics 25(16) 10.1093/bioinformatics/btp352 PMC2723002 19505943

[CR21] Mielonen O, Pratas D, Hedman K, Sajantila A, Perdomo M (2022) Detection of low-copy human virus DNA upon prolonged formalin fixation. Viruses 14(1):133 10.3390/v14010133 PMC8779449 35062338

[CR22] Nüesch JP, Lacroix J, Marchini A, Rommelaere J (2012). Molecular pathways: rodent parvoviruses–mechanisms of oncolysis and prospects for clinical cancer treatment. Clin Cancer Res : Official J Am Assoc Cancer Res.

[CR23] Phan TG, Dreno B, da Costa AC, Li L, Orlandi P, Deng X, Kapusinszky B, Siqueira J, Knol AC, Halary F, Dantal J, Alexander KA, Pesavento PA, Delwart E (2016). A new protoparvovirus in human fecal samples and cutaneous T cell lymphomas (mycosis fungoides). Virology.

[CR24] Plummer M, De Martel C, Vignat J, Ferlay J, Bray F, Franceschi S (2016). Global burden of cancers attributable to infections in 2012: a synthetic analysis. Lancet Glob Health.

[CR25] Pratas D, Hosseini M, Grilo G, Pinho AA, Silva RM, Caetano T, Carneiro J, Pereira F (2018) Metagenomic composition analysis of an ancient sequenced polar bear jawbone from Svalbard. Genes (Basel) 9(9):445 10.3390/genes9090445 PMC6162538 30200636

[CR26] Pratas D, Toppinen M, Pyöriä L, Hedman K, Sajantila A, Perdomo MF (2020) A hybrid pipeline for reconstruction and analysis of viral genomes at multi-organ level. Gigascience 9(8) 10.1093/gigascience/giaa086 PMC7439602 32815536

[CR27] Preston DL, Ron E, Yonehara S, Kobuke T, Fujii H, Kishikawa M, Tokunaga M, Tokuoka S, Mabuchi K (2002). Tumors of the nervous system and pituitary gland associated with atomic bomb radiation exposure. J Natl Cancer Inst.

[CR28] Prezioso C, Carletti R, Obregon F, Piacentini F, Manicone AM, Soda G, Moens U, Di Gioia C, Pietropaolo V (2021) Evaluation of Merkel cell polyomavirus DNA in tissue samples from Italian patients with diagnosis of MCC. Viruses 13(1) 10.3390/v13010061 PMC7824763 33466354

[CR29] Pyöriä L, Jokinen M, Toppinen M, Salminen H, Vuorinen T, Hukkanen V, Schmotz C, Elbasani E, Ojala PM, Hedman K, Välimaa H, Perdomo MF (2020) HERQ-9 is a new multiplex PCR for differentiation and quantification of all nine human herpesviruses. mSphere 5:e00265–20. 10.1128/mSphere.00265-20 10.1128/mSphere.00265-20 PMC7316487 32581076

[CR30] Qiu J, Söderlund-Venermo M, Young NS (2017). Human Parvoviruses. Clin Microbiol Rev.

[CR31] Quinlan AR (2014) BEDTools: The Swiss-Army tool for genome feature analysis. Curr Protoc Bioinformatics 47:11.12:1–34 10.1002/0471250953.bi1112s47 PMC4213956 25199790

[CR32] Rahman M, Dastmalchi F, Karachi A, Mitchell D (2018) The role of CMV in glioblastoma and implications for immunotherapeutic strategies. Oncoimmunology 8(1):e1514921 10.1080/2162402X.2018.1514921 PMC6287786 30546954

[CR33] Ron E, Modan B, Boice JD, Alfandary E, Stovall M, Chetrit A, Katz L (1988) Tumors of the brain and nervous system after radiotherapy in childhood. N Engl J Med 319(16):1033–1039 10.1056/NEJM198810203191601 3173432

[CR34] Schneider AB, Ron E, Lubin J, Stovall M, Shore-Freedman E, Tolentino J, Collins BJ (2008) Acoustic neuromas following childhood radiation treatment for benign conditions of the head and neck. Neuro Oncol 10(1):73–78 10.1215/15228517-2007-047 PMC2600840 18079359

[CR35] Söderlund-Venermo M (2019). Emerging human parvoviruses: the Rocky road to fame. Annu RevVirol.

[CR36] Syrjänen K, Syrjänen S, Lamberg M, Pyrhönen S, Nuutinen J (1983) Morphological and immunohistochemical evidence suggesting human papillomavirus (HPV) involvement in oral squamous cell carcinogenesis. Int J Oral Surg 12:418–424 10.1016/s0300-9785(83)80033-7 6325356

[CR37] Tanio S, Matsushita M, Kuwamoto S, Horie Y, Kodani I, Murakami I, Ryoke K, Hayashi K (2015). Low prevalence of Merkel cell polyomavirus with low viral loads in oral and maxillofacial tumours or tumour-like lesions from immunocompetent patients: absence of Merkel cell polyomavirus-associated neoplasms. Mol Clin Oncol.

[CR38] Toppinen M, Norja P, Aaltonen LM, Wessberg S, Hedman L, Söderlund-Venermo M, Hedman K (2015). A new quantitative PCR for human parvovirus B19 genotypes. J Virol Methods.

[CR39] Toppinen M, Pratas D, Väisänen E, Söderlund-Venermo M, Hedman K, Perdomo MF, Sajantila A (2020) The landscape of persistent human DNA viruses in femoral bone. Forensic Sci Int Genet 48:102353 10.1016/j.fsigen.2020.102353 32668397

[CR40] Väisänen E, Fu Y, Koskenmies S, Fyhrquist N, Wang Y, Keinonen A, Mäkisalo H, Väkevä L, Pitkänen S, Ranki A, Hedman K, Söderlund-Venermo M (2019). Cutavirus DNA in malignant and nonmalignant skin of cutaneous T-cell lymphoma and organ transplant patients but not of healthy adults. Clin Infect Dis: Official Publication Infect Dis Soc Am.

[CR41] Väisänen E, Kuisma I, Mäkinen M, Ilonen J, Veijola R, Toppari J, Hedman KA-O, Söderlund-Venermo MA-O (2022) Torque Teno virus primary infection kinetics in early childhood. Viruses 14(6) 10.3390/v14061277 PMC9231046 35746748

