# Supplementary figures and images for: Herpesviruses, polyomaviruses, parvoviruses, papillomaviruses, and anelloviruses in vestibular schwannoma
Source: J Neurovirol. 2023 Mar 1;29(2):226–31. doi: 10.1007/s13365-023-01112-8 (PMC10185587; doi:10.1007/s13365-023-01112-8)

**Supplemental table 1. List of viruses screened with qPCR and NGS methods**

**
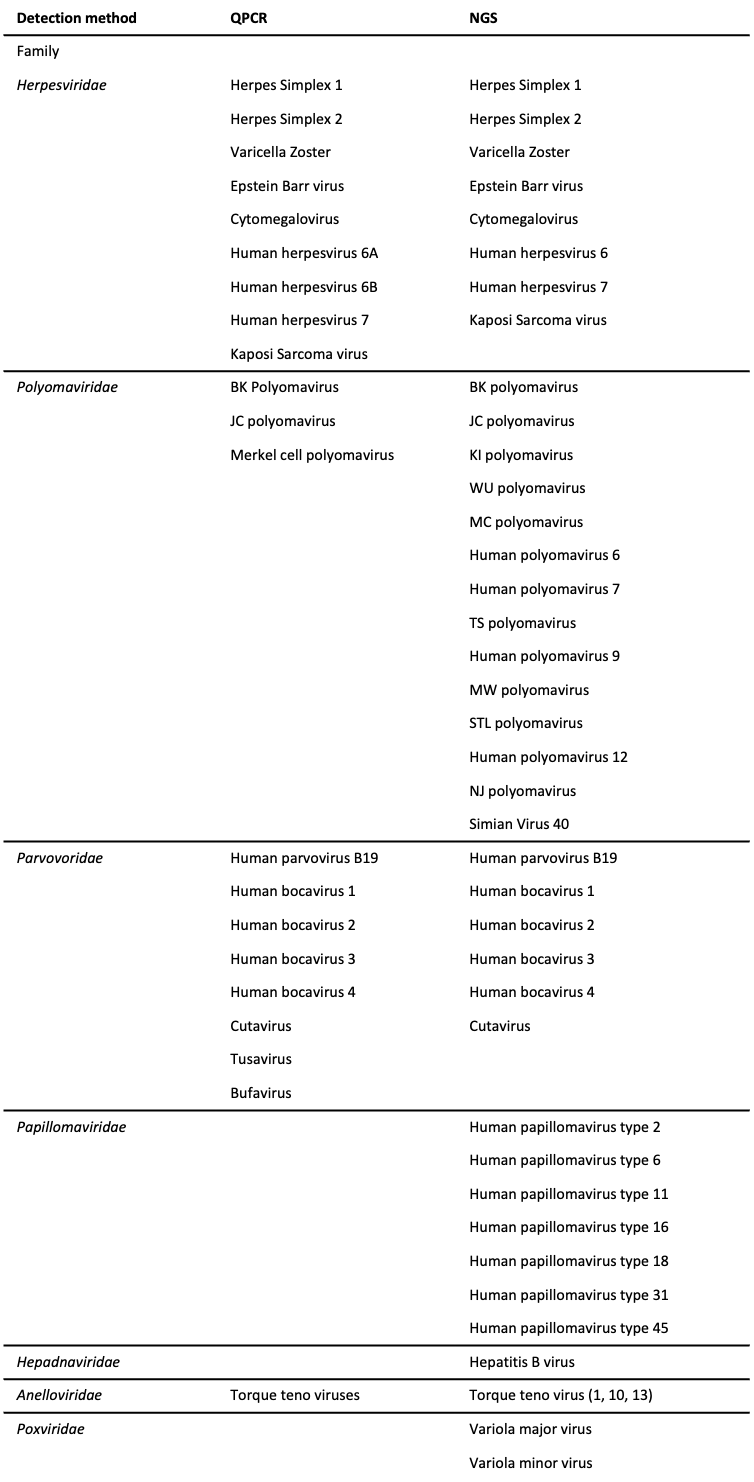
**

Supplement: Supplementary file 1 — Supplementary file1 (DOCX 135 KB) [file 13365_2023_1112_MOESM1_ESM.docx]
